# Haemoglobin A1c even within non-diabetic level is a predictor of cardiovascular disease in a general Japanese population: the Hisayama Study

**DOI:** 10.1186/1475-2840-12-164

**Published:** 2013-11-07

**Authors:** Fumie Ikeda, Yasufumi Doi, Toshiharu Ninomiya, Yoichiro Hirakawa, Naoko Mukai, Jun Hata, Kentaro Shikata, Daigo Yoshida, Takayuki Matsumoto, Takanari Kitazono, Yutaka Kiyohara

**Affiliations:** 1 Department of Environmental Medicine, Graduate School of Medical Sciences, Kyushu University, 3-1-1 Maidashi, Higashi-ku, Fukuoka City 812-8582, Japan; 2 Department of Medicine and Clinical Science, Graduate School of Medical Sciences, Kyushu University, Fukuoka, Japan

**Keywords:** Haemoglobin A1c, Cardiovascular disease, Risk factor, Prospective cohort study, Epidemiology

## Abstract

**Background:**

There is little information about predictive ability of haemoglobin A1c (HbA1c) for cardiovascular disease (CVD) in Asians. To investigate the discriminatory ability of HbA1c to identify subjects who are at greater risk of developing CVD in a prospective study of a defined community-dwelling Japanese population.

**Methods:**

A total of 2,851 subjects aged 40–79 years were stratified into five groups (HbA1c levels with ≤ 5.0, 5.1–5.4, 5.5–6.4, and ≥ 6.5% and a group with antidiabetic medication) and followed up prospectively for 7 years (2002–2009).

**Results:**

During the follow-up, 119 subjects developed CVD. The multivariable-adjusted risk of CVD was significantly increased in subjects with HbA1c levels of 5.5–6.4 and ≥ 6.5% and diabetic medication compared to HbA1c level with ≤ 5.0% (hazard ratio, 2.26 [95% confidence interval, 1.29–3.95] for the 5.5–6.4%; 4.43 [2.09–9.37] for the ≥ 6.5%; and 5.15 [2.65–10.0] for the antidiabetic medication group). With regard to CVD subtype, the positive associations between HbA1c levels and the risk of coronary heart disease (CHD) and ischaemic stroke were also significant, but no such associations were seen for haemorrhagic stroke. The C statistic for developing CVD was significantly increased by adding HbA1c values to the model including other risk factors (0.789 vs. 0762, p = 0.006), and the net reclassification improvement was 0.105 (p = 0.004).

**Conclusions:**

Our findings suggest that elevated HbA1c levels are an independent risk factor for CVD, especially CHD and ischaemic stroke, and that the addition of HbA1c to the model with traditional risk factors significantly improves the predictive ability of CVD.

## Background

Prior epidemiological studies have established that hyperglycaemia is a significant risk factor for the development of cardiovascular disease (CVD), including coronary heart disease (CHD) and stroke [1-3]. Various laboratory data are now used as indicators of hyperglycaemia, mainly including fasting plasma glucose and 2-hour postload glucose by a 75 g oral glucose tolerance test. The latter, however, is considered an expensive, complex, time-consuming method that burdens subjects with a high load. In contrast, haemoglobin A1c (HbA1c) is also widely used as a marker of average blood glucose concentrations over the preceding 2 to 3 months and has two advantages over glucose tests: it does not require subjects to fast [4], and its intraindividual variability is smaller than that of glucose measurements [5]. This background, and the basis of the association of HbA1c with diabetic retinopathy, have led the American Diabetes Association (ADA) to adopt the HbA1c level of ≥6.5% as a diagnostic criterion of diabetes [4]. However, it should be fully verified whether or not HbA1c can predict macrovascular complications. Several population-based cohort studies from Western countries have investigated the association between HbA1c and the risk of CVDs [6-11], while only a few studies from Asia have examined this issue [12-14]. Moreover, there is little information about whether or not adding HbA1c to other potential risk factors improves the ability to predict CVD [2,11,15].

The purposes of the present study were to address the associations between HbA1c levels and the development of CVD, and to investigate the discriminatory ability of HbA1c to identify subjects who are at greater risk of developing CVD in a prospective study of a defined community-dwelling Japanese population.

## Methods

### Study population and follow-up survey

A population-based prospective study of CVD and malignancy has been under way since 1961 in the town of Hisayama, a suburb of the Fukuoka metropolitan area of Kyushu Island in southern Japan. A detailed description of this survey was published previously [16]. In 2002, of a total 3,896 residents aged 40 to 79 years on the town registry, 3,000 (participation rate, 77.0%) consented to participate in the examination and underwent a comprehensive assessment for the present study. Among these, 146 who had a history of stroke or CHD based on questionnaires and medical records were excluded, as were 3 others whose HbA1c levels were not measured. The remaining 2,851 subjects (1,223 men and 1,628 women, mean age 58.8 years) were enrolled in the study.

The subjects were followed prospectively for 7 years from 2002 to 2009 by repeated health examinations. The health status was checked yearly by mail or telephone if subjects did not undergo a regular examination or had moved from town. We also established a daily monitoring system among the study team, local physicians, and members of the town’s Health and Welfare Office. Using this system, we gathered information on new CVD events, including suspected cases. When stroke or CHD occurred or was suspected, physicians in the study team examined the subject and evaluated his or her detailed clinical information. In addition, when a subject died, an autopsy was performed at the Departments of Pathology of Kyushu University. During the follow-up period, none of the subjects were lost to follow-up, and 144 subjects died, of whom 95 (66.0%) underwent autopsy.

### Clinical evaluation and laboratory measurements

At the baseline examination, HbA1c was measured by latex aggregation immunoassay using Determiner HbA1C (Kyowa Medix, Tokyo, Japan). The value for HbA1c was estimated as a National Glycohemoglobin Standardization Program equivalent value calculated with the formula [17]: HbA1c (%) = 1.02 × HbA1c (Japan Diabetes Society) (%) + 0.25%. The 75 g oral glucose tolerance test was performed for 2,412 subjects in a fasting state. Plasma glucose levels were determined by a glucose-oxidase method. Total and high-density lipoprotein (HDL) cholesterol concentrations were measured by an enzymatic autoanalyzer.

Blood pressure was obtained three times using an automated sphygmomanometer (BP-203RV III; Colin, Tokyo, Japan) with the subject in a sitting position after resting for at least 5 minutes. The mean value of the three measurements was used for the analysis. Hypertension was defined as blood pressure ≥140/90 mmHg and/or current use of antihypertensive agents. Height and weight were measured with the subject in light clothes without shoes, and body mass index (BMI) was calculated (kg/m^2^). Electrocardiogram (ECG) abnormalities were defined as left ventricular hypertrophy (Minnesota Code 3–1), ST depression (4–1, 2, 3), or atrial fibrillation (8–3). Each participant completed a self-administered questionnaire covering lifestyles, medical histories including antidiabetic treatment. Smoking habits and alcohol intake were classified as either current use or not. Subjects engaging in sports or other forms of exercise ≥3 times a week during their leisure time were included in the physically active group.

### Definition of cardiovascular events

CVD was defined as a first-ever occurrence of CHD or stroke. The criteria for a diagnosis of CHD included acute myocardial infarction, silent myocardial infarction, sudden cardiac death within 1 hour after the onset of acute illness, and coronary artery disease treated by coronary artery bypass surgery or angioplasty. Acute myocardial infarction was diagnosed when a subject met at least two of the following criteria: (1) typical symptoms, including prolonged severe anterior chest pain; (2) evolving diagnostic electrocardiographic changes; (3) cardiac enzyme levels more than twice the upper limit of normal range; (4) morphological changes, including local asynergy of cardiac wall motion on echocardiography, persistent perfusion defect on cardiac scintigraphy, or myocardial necrosis or scars ≥1 cm long accompanied by coronary atherosclerosis at autopsy. Silent myocardial infarction was diagnosed for participants who had no historical indication of clinical symptoms or abnormal cardiac enzyme changes according to either of two criteria: (1) New onset of abnormal Q waves on ECG plus morphological myocardium changes (local asynergy on echocardiography or persistent perfusion defect on scintigraphy), or (2) myocardial necrosis or scars ≥1 cm long accompanied by coronary atherosclerosis at autopsy. Stroke was defined as a sudden onset of nonconvulsive and focal neurological deficit persisting for ≥24 hours. The diagnosis and classification of stroke were determined on the basis of clinical information, including brain CT/MRI or autopsy findings. Stroke was classified as either ischaemic or haemorrhagic.

### Statistical analysis

We classified all of the subjects into 5 groups on the basis of baseline information on antidiabetic medication and HbA1c levels. We first divided the subjects into 2 groups according to use of antidiabetic medication. Among the subjects without antidiabetic medication, those with HbA1c levels of ≥6.5% were considered as a group having diabetes according to the ADA guidelines, and those with levels of <6.5% were further divided into tertiles by HbA1c levels (≤5.0, 5.1–5.4, and 5.5–6.4%). The incidence of CVD and its subtypes was calculated by the person–year method and adjusted for age and sex by the direct method using 10-year age groupings of the overall study population. The age- and sex- and multivariable-adjusted hazard ratios (HRs) and their 95% confidence intervals (CIs) were estimated using the Cox proportional hazards model. To compare the discrimination of incident CVD between the models adjusted for known CVD risk factors with and without HbA1c, C statistics analogous to the area under the receiver operating curve were estimated. The statistical significance of differences was compared using the method of DeLong et al. [18]. Moreover, the increased discriminatory value of HbA1c levels was further examined by the net reclassification improvement (NRI) and integrated discrimination improvement (IDI) [19]. The NRI evaluates changes in estimated prediction probabilities that imply a change from one category to another between different models. In this analysis, we classified the probability of the risk of CVD for 7 years into three categories of <2.0%, 2.0 to 4.0%, and >4.0%, referring to the median values of the predicted probabilities among the participants with and without incident CVD. Continuous NRI values were also estimated using the predicted probabilities taken as continuous variables. The IDI considers differences in discrimination slopes between different models, where the discrimination slope was defined as the difference between the mean of the estimated prediction probabilities taken as continuous variables for individuals with events and the corresponding mean for those without events. The proportions of missing values were less than 0.1% for all the variables included in the model. A two-sided p < 0.05 value was considered statistically significant in all analyses. Because there was no interaction between sex and HbA1c levels (data not shown), we included men and women together in all analyses. Statistical analyses were conducted using Statistical Analysis Software (SAS) version 9.3 (SAS Institute, Cary, NC).

### Ethical considerations

The study protocol was approved by the Kyushu University Institutional Review Board for Clinical Research, and the procedures followed were in accordance with national guidelines. All participants provided written informed consent.

## Results

Table 1 compares the mean values or frequencies of possible risk factors for CVD among the HbA1c levels and antidiabetic medication. The mean values of age, systolic and diastolic blood pressures, BMI, and total cholesterol and frequencies of hypertension were increased with increasing HbA1c levels, whereas the mean HDL cholesterol values and frequencies of smoking habits and alcohol intake decreased. The frequencies of men, ECG abnormalities, and regular exercise did not differ across HbA1c levels. The subjects with antidiabetic medication had higher mean (± SD) HbA1c (7.3 ± 1.4%) than the rest of the study subjects (5.3 ± 0.7%). The former were older, and had higher frequencies of men and hypertension.

**Table 1:** Baseline characteristics of subjects by the haemoglobin A1c levels and group with antidiabetic medication

	**HbA1c level (%)**				**Antidiabetic medication**
	**≤ 5.0**	**5.1–5.4**	**5.5–6.4**	**≤ 6.5**	
**Variables**	**(n = 955)**	**(n = 923)**	**(n = 736)**	**(n = 104)**	**(n = 133)**
Age (years)	56 (11)	59 (10)	61 (10)	60 (10)	65 (9)
Men (%)	46.0	38.1	41.9	47.1	56.4
Fasting plasma glucose (mmol/L)*	5.6 (0.5)	5.8 (0.5)	6.2 (0.8)	9.2 (2.6)	8.7 (2.0)
2-hour postload glucose (mmol/L)*	6.5 (1.5)	7.1 (1.9)	8.6 (3.1)	16.9 (5.3)	17.1 (4.7)
Systolic blood pressure (mmHg)	128 (20)	129 (19)	135 (21)	143 (22)	139 (22)
Diastolic blood pressure (mmHg)	77 (12)	78 (12)	80 (12)	84 (13)	81 (12)
Hypertension (%)	33.5	35.8	49.3	54.8	69.9
ECG abnormalities (%)	15.5	12.2	17.7	18.3	14.3
Body mass index (kg/m^2^)	22.4 (2.9)	23.3 (3.2)	24.0 (3.6)	25.8 (4.3)	23.8 (3.4)
Total cholesterol (mmol/l)	5.12 (0.90)	5.37 (0.90)	5.41 (0.94)	5.62 (0.91)	5.29 (0.94)
HDL cholesterol (mmol/l)	1.70 (0.44)	1.62 (0.41)	1.56 (0.39)	1.46 (0.39)	1.58 (0.42)
Current smoking (%)	27.2	22.0	20.2	26.0	18.8
Current alcohol use (%)	52.7	44.0	39.3	48.1	44.4
Regular exercise (%)	10.6	8.8	12.8	8.7	15.8

*Abbreviations*: HbA1c, Haemoglobin A1c; ECG, Electrocardiogram; HDL, High-density lipoprotein.

All values are given as means (SD) or as percentages.

*These parameters were measured in only 2,412 subjects who underwent a 75 g oral glucose tolerance test.

**Figure 1:**
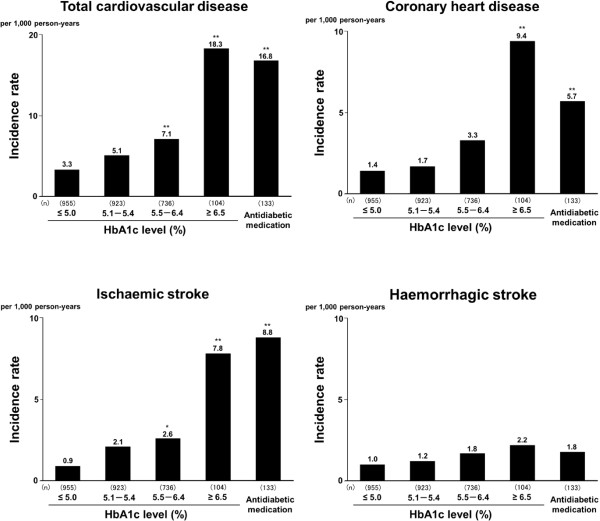
**Age- and sex-adjusted incidence of cardiovascular disease by haemoglobin A1c levels and antidiabetic medication.** Abbreviations: HbA1c, haemoglobin A1c. * p < 0.05, ** p < 0.01 vs. HbA1c 5.0%.

During the 7-year follow-up, we identified 119 first-ever CVD events (75 men and 44 women). Of these, there were 48 CHD events (37 men and 11 women; 29 acute myocardial infarction, 4 silent myocardial infarction, 3 sudden cardiac death within 1 hour after the onset of acute illness, and 12 coronary artery disease treated by coronary artery bypass surgery or angioplasty), 46 ischemic stroke events (24 men and 22 women), and 29 haemorrhagic stroke events (17 men and 12 women). Figure 1 shows the age- and sex-adjusted incidence of CVD and its subtypes according to the HbA1c levels and antidiabetic medication. The incidence of total CVD increased significantly with rising HbA1c levels: there were significant differences between subjects with HbA1c level of ≤5.0% and those with HbA1c of 5.5–6.4% or higher (p < 0.01). A similar pattern was observed for the age- and sex-adjusted incidences of CHD and ischaemic stroke, but no such association was seen for haemorrhagic stroke.

As shown in Table 2, these associations were substantially unchanged even after controlling for the confounding factors: age, sex, hypertension, ECG abnormalities, BMI, total and HDL cholesterol, smoking habit, alcohol intake, and regular exercise (CVD: HbA1c 5.5–6.4%, multivariable-adjusted HR = 2.26 [95% CI 1.29–3.95], p = 0.004; ≥6.5%, HR = 4.43 [95% CI 2.09–9.37], p = <0.001; CHD: 5.5–6.4%, HR = 2.11 [95% CI 0.90–4.95], p = 0.09; ≥6.5%, HR = 3.55 [95% CI 1.11–11.3], p = 0.03; ischaemic stroke, 5.5–6.4%, HR = 3.57 [95% CI 1.27–10.0], p = 0.02; ≥6.5%, HR = 9.65 [95% CI 2.81–33.1], p < 0.001).

**Table 2:** Adjusted hazard ratios for cardiovascular disease by the haemoglobin A1c level and group with antidiabetic medication

	**HbA1c level**							**Antidiabetic medication**	
	**≤ 5.0**	**5.1–5.4**		**5.5–6.4**		**≤ 6.5**			
No. of subjects at risk	955	923		736		104		133	
Total cardiovascular disease (n)	19	31		39		12		18	
Age- and sex-adjusted HR (95% CI)	1.00	1.56	(0.88–2.78)	2.19	(1.26–3.81)^b^	4.94	(2.39–10.2)^b^	4.53	(2.36–8.69)^b^
Multivariable-adjusted HR (95% CI)	1.00	1.60	(0.90–2.85)	2.26	(1.29–3.95)^b^	4.43	(2.09–9.37)^b^	5.15	(2.65–10.0)^b^
Coronary heart disease (n)	8	10		17		5		8	
Age- and sex-adjusted HR (95% CI)	1.00	1.24	(0.49–3.16)	2.28	(0.98–5.31)	4.63	(1.51–14.2)^b^	4.30	(1.61–11.5)^b^
Multivariable-adjusted HR (95% CI)	1.00	1.15	(0.45–2.93)	2.11	(0.90–4.95)	3.55	(1.11–11.3)^a^	4.39	(1.60–12.0)^b^
Ischaemic stroke (n)	5	13		15		6		7	
Age- and sex-adjusted HR (95% CI)	1.00	2.42	(0.86–6.82)	3.19	(1.15–8.83)^a^	9.47	(2.88–31.1)^b^	6.99	(2.18–22.4)^b^
Multivariable-adjusted HR (95% CI)	1.00	2.57	(0.91–7.29)	3.57	(1.27–10.0)^a^	9.65	(2.81–33.1)^b^	8.33	(2.54–27.3)^b^
Haemorrhagic stroke (n)	6	8		10		2		3	
Age- and sex-adjusted HR (95% CI)	1.00	1.26	(0.44–3.66)	1.77	(0.64–4.91)	2.58	(0.52–12.8)	2.38	(0.58–9.66)
Multivariable-adjusted HR (95% CI)	1.00	1.42	(0.48–4.14)	1.87	(0.66–5.25)	2.41	(0.46–12.5)	2.70	(0.65–11.3)

*Abbreviations*: HbA1c, Haemoglobin A1c; HR, Hazard ratio; CI, Confidence interval.

^a^p < 0.05, ^b^p < 0.01 vs. Haemoglobin A1c ≤5.0%.

Multivariable adjustment was made for age, sex, hypertension, electrocardiogram abnormalities, body mass index, total and high-density lipoprotein cholesterol levels, current smoking, current alcohol use, and regular exercise.

Finally, to evaluate the impact of HbA1c on the accuracy of CVD risk assessment, we compared the discriminatory abilities between models with and without HbA1c. In this analysis, we included all subjects, because there was no interaction between use of antidiabetic medication and HbA1c levels (data not shown). The C statistic for a model including other potential risk factors, the aforementioned items, was 0.762, and increased significantly more with the addition of a continuous HbA1c value (C statistics = 0.789, p = 0.006). Furthermore, the cross-tabulation for the absolute risk of CVD development between the models with and without HbA1c values is shown in Table 3. When the basic model with HbA1c levels was used, 10 subjects were correctly reclassified into a higher risk category, whereas 8 subjects were inappropriately reclassified into a lower risk category among subjects who had developed CVD. On the other hand, 331 subjects were correctly reclassified into a lower risk category, and 91 subjects were inappropriately reclassified into a higher risk category among subjects who had not developed CVD. In addition, the three-category NRI was estimated as 0.105 (Z_NRI_ =2.87, p = 0.004), the continuous NRI was 0.356 (Z_NRI_ =3.80, p < 0.001), and the IDI was calculated as 0.021 (Z_IDI_ = 2.88, p = 0.004).

**Table 3:** Reclassification for the absolute risk of cardiovascular disease development in the Hisayama study, 2002-2009


Number of subjects who developed cardiovascular disease				
	Basic model + HbA1c			
Basic model	<2.0%	2.0–4.0%	>4.0%	Total
<2.0%	7	3	2	12
2.0–4.0%	2	15	5	22
>4.0%	0	6	79	85
Total	9	24	86	119
Number of subjects who did not develop cardiovascular disease				
	Basic model + HbA1c			
Basic model	<2.0%	2.0–4.0%	>4.0%	Total
<2.0%	1,065	30	5	1,100
2.0–4.0%	170	488	56	714
>4.0%	0	161	756	917
Total	1,235	679	817	2,731

*Abbreviations*: HbA1c, Haemoglobin A1c.

The basic model included age, sex, hypertension, electrocardiogram abnormalities, body mass index, total and high-density lipoprotein cholesterol levels, current smoking, current alcohol use, and regular exercise.

In addition, in a subgroup analysis we compared the predictive ability between HbA1c and fasting plasma glucose or 2-hour postload glucose to identify the individuals at high risk of future CVD among 2,412 subjects who had undergone a 75 g oral glucose tolerance test. However, no statistical significance was found between them (data not shown).

## Discussion

In a prospective study of a general Japanese population, we clearly demonstrated that elevated HbA1c levels, even in the prediabetic range, were a significant risk factor for the development of CVD, especially for CHD and ischaemic stroke, in a general Japanese population. These associations remained robust even after controlling for comprehensive confounding factors. Furthermore, adding the HbA1c value to the confounding factors significantly improved the predictive ability for total CVD. These findings are important in that they indicate HbA1c’s value for predicting the long-term risk of CVD in Japanese.

Some clinical and population-based cohort studies have shown that increased HbA1c levels were positively associated with the risks of CVDs and mortality [6-11,20-25]. In the present study, the risks of CHD and ischaemic stroke were significantly or marginally higher even in the subjects with HbA1c levels of ≥5.5% compared with those with HbA1c levels of ≤5.0%. There has been controversy over whether or not the prediabetic levels of HbA1c are associated with CVD risk. Prior cohort studies in Caucasian populations have shown positive associations between the prediabetic range of HbA1c levels and the incidence of total CVD or CHD [6,7,9,11,26]. A clinical study from Japan has revealed that HbA1c is significantly associated with the complexity of coronary lesions even in non-diabetic adults [27]. Likewise, such an association was observed for ischaemic stroke in other cohort studies [8,11,12]. Our findings are in accordance with these studies. On the other hand, some prospective studies have demonstrated that the risk of CVD was increased only in subjects with diabetic levels of HbA1c and not in those with prediabetic levels [10,13,28]. This inconsistency in findings might be caused by differences in population and methodology among the studies.

Contrary to the studies reporting an association between HbA1c levels and the risk of CHD or ischaemic stroke, investigations on haemorrhagic stroke have been scarce. One prospective study from Japan assessed the association between HbA1c levels and haemorrhagic stroke incidence, and found no significant association [12]. In the present study, the association of HbA1c levels with the risk of haemorrhagic stroke was also not significant. These findings suggest that hyperglycaemia scarcely affects the aetiology of haemorrhagic stroke, which is generally recognized as the disruption of a vessel wall [29]. However, the number of haemorrhagic stroke events was limited in these studies. Therefore, further epidemiological research is required to elucidate this issue.

To the best of our knowledge, this is the first cohort study from Asia to demonstrate the predictive ability of HbA1c to identify persons at high risk of developing CVD. The Framingham Study showed that the addition of glycaemic categories did not substantially improve the ability to predict CVD beyond comprehensive CVD risk factors alone [2]. However, two recent cohort studies reported opposite results. The addition of HbA1c to the Framingham risk score made a small but statistically significant improvement to the ability to discriminate CHD in men in the European Prospective Investigation of Cancer - Norfolk cohort [15]. The Atherosclerosis Risk in Communities Study also demonstrated that NRI and IDI for CHD were significantly improved with the addition of HbA1c to the model of fasting plasma glucose and other covariates [11]. These findings, when taken together with ours, indicate it is likely that adding HbA1c to other comprehensive risk factors will improve future discriminatory ability for CVD. Since few studies have investigated this issue, further research is expected.

Why does HbA1c have the potential to predict CVD? Complex and diverse hypotheses have been proposed to explain the causal relationship between hyperglycaemia with atherosclerosis, and one of the important pathways is recognized as glycosylation. Glycosylation is a non-enzymatic reaction induced by chronic hyperglycaemia, and is process *in vivo* results in two different products: early and advanced glycation end products (AGEs). HbA1c is well known as one of the early glycation end products and is a precursor of AGEs [30,31]. It was reported that AGEs decreased large-vessel elasticity and induced inflammatory and pro-thrombotic responses in the vessel wall, thereby being involved in vascular complications [32,33]. Some clinical studies demonstrated that high AGEs levels were associated with the risk of CVD [34,35]. Therefore, the biological mechanisms of glycosylation may be one of the reasons for the relationship between HbA1c and the risk of CVD observed in our study.

In the present study, the linear association between HbA1c levels and the risk of incident CVD was observed. There is a debate as to whether or not the low-normal glycaemic state determined by HbA1c is associated with increased CVD risk. At least five studies have shown linearly increasing risks of CVD with higher HbA1c [2,9,36-38]. In contrast, other studies showed a U- or J-shaped curve for the risk of CVD incidence and total or CVD death [10,11,39,40]. This discrepancy might result from differences in study design, outcomes, and race or ethnicity. Further exploration of the health risks associated with the low-normal glycaemic state derived from HbA1c is warranted.

The strengths of our study include its longitudinal population-based design, low selection bias at baseline, perfect follow-up of subjects, and accuracy of diagnosis of CVD. However, some limitations of our study should be discussed. First, HbA1c and other potentially confounding factors were based on a single measurement at baseline, although this limitation is typical of most prospective studies. During the follow-up, risk factor levels changed due to modifications in lifestyle or medication, and misclassification of these levels was possible. This could have weakened the association found in this study, biasing the results toward the null hypothesis. Thus, the true association may be stronger than that shown in our study. Second, our study included the relatively small number of event cases of CVD, consisting of stroke and CHD. The cardiovascular spectrum ranges much further, including congestive heart failure, transient ischaemic attack, and peripheral artery disease. If the CVD range would be extended with these diseases, the number of events would increase, and this would solve the power issue. Unfortunately, however, we could not include congestive heart failure, transient ischaemic attack, or peripheral artery disease in the endpoints, because we do not have information on events of these diseases. Last, our study population was comprised of one ethnic group, and thus, generalizability to other ethnicities may be limited. On the other hand, the use of a monoethnic group in such a study avoids problems relevant to population stratification artifacts. Further studies in other ethnic groups will be needed to determine the applicability of HbA1c levels to the prediction of vascular events.

## Conclusions

A higher level of HbA1c was an independent risk factor for CVD, especially CHD and ischaemic stroke, in a general Japanese population. Moreover, we showed that the measurement of HbA1c improves the discriminatory ability of CVD. Further investigations are required to clarify the association between HbA1c and the type of CVD, especially in Asian countries where the number of diabetic individuals has been rapidly increasing.

## Abbreviations

HbA1c: Haemoglobin A1c; CVD: Cardiovascular disease; CHD: Coronary heart disease; ADA: American Diabetes Association; HDL: High-density lipoprotein; BMI: Body mass index; ECG: Electrocardiogram; CT: Computed tomography; MRI: Magnetic resonance imaging; HR: Hazard ratio; CI: Confidence interval; NRI: Net reclassification improvement; IDI: Integrated discrimination improvement; AGE: Advanced glycation end product; SD: Standard deviation.

## Competing interests

The authors declare that they have no competing interests.

## Authors’ contributions

FI conceived and designed the study, and was responsible for acquisition of data, statistical analysis, and drafting the manuscript. YD contributed to acquisition and interpretation of data and drafting of the manuscript. TN, YH, NM, JH, KS, and DY collected data and provided critical review of the draft. TM and TK reviewed the manuscript. YK contributed to interpretation of data and drafting manuscript, obtained funding, and was a study supervisor. All authors read and approved the final manuscript.
